# Never injected, but hepatitis C virus-infected: a study among self-declared never-injecting drug users from the Amsterdam Cohort Studies

**DOI:** 10.1111/j.1365-2893.2009.01105.x

**Published:** 2009-08

**Authors:** C H S B van den Berg, T J W van de Laar, A Kok, F R Zuure, R A Coutinho, M Prins

**Affiliations:** 1 Department of Experimental Virology, Center for Infection and Immunology Amsterdam (CINIMA), Academic Medical CenterAmsterdam, The Netherlands; 2 Cluster Infectious Diseases, Department of Research, Amsterdam Health ServiceAmsterdam, The Netherlands; 3 Cluster Infectious Diseases, Laboratory of Public Health, Amsterdam Health ServiceAmsterdam, The Netherlands; 4 Center for Infectious Disease Control, National Center for Public Health and the EnvironmentBilthoven, The Netherlands; 5 Department of Internal Medicine, Division of Infectious Diseases, Tropical Medicine and AIDS, CINIMA, Academic Medical CenterAmsterdam, The Netherlands

**Keywords:** cohort study, hepatitis C virus, noninjecting drug users

## Abstract

The aim of this study was to gain insight in transmission routes of hepatitis C virus (HCV) infection among never-injecting drug users (DU) by studying, incidence, prevalence, determinants and molecular epidemiology of HCV infection. From the Amsterdam Cohort Studies among DU, 352 never-injecting DU were longitudinally tested for HCV antibodies. Logistic regression was used to identify factors associated with antibody prevalence. Part of HCV NS5B was sequenced to determine HCV genotype and for phylogenetic analyses, in which sequences were compared with those from injecting DU. HCV antibody prevalence was 6.3% and HCV incidence was 0.49/1000 PY. HIV-positive status, female sex and starting injection drug use during follow-up (a putative marker of past injection drug use), were independently associated with HCV prevalence. The main genotypes found were genotype 3a (50%) and 1a (30%). Phylogenetic analysis revealed that HCV strains in never-injecting DU did not cluster together and did not differ from HCV strains circulating in injecting DU. We found a higher HCV prevalence in never-injecting DU than in the general population. Phylogenetic analysis shows a strong link with the injecting DU population. The increased risk could be related to underreporting of injecting drug use or to household or sexual transmission from injectors to noninjectors. Our findings stress the need for HCV testing of DU who report never injecting, especially given the potential to treat HCV infection effectively.

## Introduction

Acute hepatitis C virus (HCV) infection is usually asymptomatic, and leads to chronic infection in 50–80% of patients [1]. Decades of chronic HCV infection can lead to liver cirrhosis and, in 1–5% of these patients, eventually to hepatocellular carcinoma as well [2]. In recent years, treatment success rates have substantially improved [3]. The most important mode of HCV transmission is through exposure to infected blood [1,4], and although sexual and household transmission have been described, they appear to happen only occasionally [5–7]. While never-injecting drug users (DU) do not share needles and/or syringes, their HCV prevalence is still higher than in the general population. Some studies suggest that HCV infection in never-injecting DU is associated with the sharing of drug-use paraphernalia, especially utilities used for consumption of crack, but others could not confirm these findings (reviewed in Ref. [8]) Alternatively, never-injecting DU might become infected with HCV through needle-stick accidents, household transmission, or sexual exposure. Recent review of research describing HCV among noninjecting DU points to a substantial gap in our knowledge of HCV in never-injecting DU, as no uniform risk factors could be identified [8].

The Amsterdam Cohort Study (ACS) among DU comprises a large group of never-injecting DU. It was designed to evaluate the sexual and blood borne transmission of HIV, other blood borne pathogens, and sexually transmitted diseases, as well as the determinants of transition to injecting drug use. This design has the potential to determine prevalence, incidence and risk factors for HCV infection among never-injecting DU. Additionally, we used phylogenetic analysis to investigate whether HCV strains isolated from never-injecting DU were closely related to strains circulating among injecting DU, or whether separate introductions had occurred through unrelated modes of transmission [9].

## Materials and methods

The ACS among DU is an open, prospective cohort study initiated in 1985 [9]. Participation in the ACS is voluntary, and informed consent is obtained for every participant at intake. Recruitment is ongoing and in recent years has been directed in particular towards young DU. Both injecting and noninjecting DU are included and visit the Amsterdam Health Service every 4–6 months. Each study visit standardized questionnaires on (injecting) drug use and sexual risk behaviour are administered by trained research nurses and blood is drawn for prospective HIV testing and storage of serum. To study HCV prevalence and incidence we retrospectively tested stored serum from all participants having at least two visits between December 1985 and November 2005 (*n* = 1276), using the first available sample in each case. Individuals who were HCV-negative at ACS entry were tested for HCV antibodies at their last ACS visit before November 2005. On finding HCV seroconversion (defined as the presence of HCV antibodies in a previously seronegative individual), we tested samples taken between these two visits to determine the moment of seroconversion (defined as the midpoint between the last HCV seronegative sample and the first seropositive visit) [10]. Third generation commercial microparticle EIA system tests were used to detect HCV antibodies (AxSym HCV version 3.0; Abbott, Wiesbaden, Germany). 28.9% of the seropositive participants were tested at two study visits or more, all with consistent positive HCV antibody test results. Presence of HCV antibodies in all never-injecting DU was confirmed with Western blot (Deciscan HCV Plus immunoblot; BioRad, Marnes la Coquette, France). All ACS samples were stored at −80 °C.

All ACS participants since 1985 (*n*=1640) were tested for HIV antibodies by enzyme-linked immunosorbent assays (ELISA), since 2003 (AxSym HIV Ag/Ab Combo, Abbott, Chicago, IL, USA), at each study visit. Results were confirmed by Western blot, since 1986, by HIV Blot version 2.2 (Genelab Diagnostics, pte Ltd, Singapore Science Park, Singapore).

### Statistical analysis

Anti-HCV antibody prevalence and incidence were calculated. Follow-up time was calculated from HCV-negative study entry through HCV seroconversion, the moment of starting injecting drug use, or November 2005, whichever occurred first.

Risk factors for the presence of HCV antibodies at study entry were examined using logistic regression. All risk factors refer to the past 6 months, unless stated otherwise. They included: general and demographic factors (sex, nationality, ethnicity, calendar year of visit); drug use-related risk factors (ever injecting drug use, years of regular heroin/cocaine/amphetamines use, start of injecting drug use during ACS follow up, alcohol use) and specifically cocaine-use-related factors (years of regular cocaine use/cocaine snorting/basing of cocaine); sexual risk behaviour (having sex with injecting DU/commercial sex workers/men who have sex with men since 1980, main sexual preference since 1980, number of commercial sexual contacts since 1980, having a steady sexual partner, having an injecting steady sexual partner, HIV status of the steady sexual partner, condom use (with steady sexual partner/casual partner/commercial contacts) and other clinically relevant variables (subjects’ history of HIV, jaundice, blood transfusion, tattoo, piercing).

Multivariate logistic regression models were built using forward stepwise techniques. All variables with a *P*-value ≤0.10 in univariate analysis were considered for entry into the model. Statistical analysis was performed by use of stata (version 9.2; StataCorp, Collage Station, Texas, USA) and spss (version 15.0; SPSS Inc., Chicago, IL, USA) software. All statistical tests were two-sided; a *P*-value ≤0.05 was considered to be statistically significant. Interaction and confounding were checked between the variables in the final models and all variables with a univariate *P*-value ≤0.20.

### Reverse-transcription polymerase chain reaction (RT-PCR) methods

After HCV antibody screening, HCV-seropositive samples were additionally tested for the presence of HCV RNA. RNA isolation was performed on 100 μL of serum using the TriPure method (Roche Diagnostics, Almere, the Netherlands). Each RNA isolate was used as input for two nested multiplex RT-PCRs. The first PCR, which targets the conserved HCV core region, was devised as a genotyping system to differentiate genotypes 1a, 1b, 2a, 2b, 3a, 4, 5a and 6a. The second RT-PCR, which targets the NS5B region, was used for phylogenetic analysis. Conditions and primers for both PCRs have been described elsewhere [11].

### Sequencing and phylogenetic analysis

The sequencing reaction and analysis were performed as described earlier [11]. Briefly, NS5B PCR products were ethanol precipitated. Sense and antisense strands were separately cycle-sequenced using the BigDye Terminator system (version 1.1; Perkin Elmer, Monza, Italy). Sequence products were purified using DyeEx spin kits (Qiagen, Hilden, Germany) and analysed on an ABI-310 automated sequencer (Applied Biosystems, Nieuwekerk aan de IJssel, the Netherlands). Sequence alignment of the 436-bp NS5B fragment was performed using the BioEdit software package [12]. Viral genotype was confirmed after phylogenetic analysis of the NS5B sequences obtained from subjects (GenBank accession numbers EU410492 to EU410507) along with established GenBank reference sequences [13] Mega software (version 3.1; available at: http://www.megasoftware.net) was used to construct a phylogenetic tree by the neighbour-joining method, using the Tamura-Nei substitution model with γ-distribution (α = 0.40). Bootstrap values (*n*=1000) were calculated to analyse the stability of tree topology. HCV sequences obtained from DU who reported never injecting were compared to all known HCV sequences from injecting DU participating in the ACS [11,14] (and unpublished data).

## Results

### General characteristics

Among the 1276 DU who participated in the ACS and had two or more visits between December 1985 and November 2005, 364 DU reported never having injected drugs before study entry. Of these 364, 352 (96.7%) had serum available for HCV testing. They were mainly male (69.3%) and of Dutch nationality (305/352, 86.6%); of the 305 Dutch participants 101 (33.1%) were of Surinamese ethnicity. Of 352 never-injecting DU, 154 preferred cocaine as their main type of noninjected drugs (43.8%). Of the 352, 22 (6.3%, 95% CI 3.9–9.4%) were HCV antibody-positive at study entry and 14/352 (4.0%, 95% CI 2.2–6.6%) DU were HIV-positive (Table 1). The total HCV-negative and never-injecting follow-up time was 2005 person years (PY); the median follow-up time per participant was 6.4 years [interquartile range (IQR) 3.01–11.3 years]. Only one never-injecting DU seroconverted for HCV during follow up; the HCV incidence was 0.049 per 100 PY (95% CI 0.01–0.35 per 100 PY). However, 47 never-injecting DU started injecting during follow-up, of whom seven were HCV-positive at study entry and 23 seroconverted for HCV after starting injection.

**Table 1:** General characteristics of never-injecting drug users (DU) at entry in the Amsterdam Cohort Studies among DU

	HCV + *n*=22	HCV −*n*=330
**General drug use and HCV related characteristics**		
Median age (IQR)	30 (26–37)	30 (26–36)
Female sex	12/22 (54.4%)	96/330 (29.1%)
Dutch nationality	19/22 (86.4%)	286/330 (86.7%)
Homeless in the past 6 months	0/14 (0%)	45/262 (17.2%)
Main type of drug used in the past 6 months		
Heroin	6/20 (30%)	137/300 (45.7%)
Cocaine	13/20 (65%)	141/300 (47%)
Heroin and cocaine together	1/20 (5%)	15/300 (5%)
Other	–	7/300 (2.3%)
HIV-positive (%)	3/22 (13.6%)	11/330 (3.33%)
Ever tattoo	6/14 (43%)	91/194 (47%)
Ever piercing	2/14 (14%)	20/194 (10%)
Jaundice (ever)	2/8 (25%)	4/68 (6%)
Blood transfusion (ever)	2/8 (25%)	5/67 (7.5%)
**Follow-up characteristics**		
Median number of visits to ACS (IQR)	15 (6–25)	12 (5–22)
Median years follow up in ACS (IQR)	7.58 (4.58–14.1)	6.13 (2.99–11.1)
Number of HCV seroconversions	–	1
**HCV viral characteristics**		
HCV RNA+	15 (68%)	NA
*Genotypes mainly related to injecting drug use*		
1a	4 (26.7%)*	
3a	8 (53.3%)*	
*Genotypes mainly related to other risks*		
1b	2 (13.3%)*	
2a	1 (6.7%)*	

NA, not applicable; *% among all HCV RNA positive individuals.

In addition to the observed HCV incidence, we calculated an estimated incidence using prevalence data, assuming that the duration of regular hard-drug use before study entry equals the time of exposure to HCV. Information on the number of years of regular cocaine/regular heroin use was available for 285/352 individuals (81.0%), including 20/22 HCV positive never-injecting DU. The duration of regular use of heroin or cocaine was used as the time of exposure. These 285 individuals had a total of 2539 PY of regular drug use. The estimated time of HCV infection was defined as the midpoint of years of duration of regular use of hard drugs, yielding an estimated incidence of 0.79 per 100 PY. Assigning the estimated time of HCV infection to either the start of regular hard drug use before study entry (maximum estimated HCV incidence) or at study entry (minimum estimated HCV incidence), changed the estimated HCV incidence only slightly to 0.82 or 0.76 per 100 PY, respectively.

### Associations with the presence of HCV antibodies

In univariate logistic regression (Table 2), the following variables were significantly associated with the presence of HCV antibodies at entry in the ACS: female sex (OR 2.93, 95% CI 1.22–7.00) and starting injection during follow-up, a putative marker of past injection drug use (OR 3.38, 95% CI 1.30–8.80). Although the association had only borderline significance, HIV-positive participants had a higher risk of being HCV-positive (OR 4.58, 95% CI 1.18–17.8, *P*=0.053) (Table 2). No significant association of HCV with crack use was found, although the OR for cocaine compared to heroin as the main type of drug used was 2.11 (95% CI 0.78–5.70) and the OR for one or more times daily cocaine use was higher compared to less frequent cocaine use in the 6 months preceding ACS entry.

**Table 2:** Univariate and multivariate logistic regression. Determinants of HCV in never-injecting drug users (DU) at entry in the Amsterdam Cohort Studies among DU

		Univariate			Multivariate		
	Proportion HCV+	OR	95% CI	*P*-value	OR	95% CI	*P*-value
**Demographic variables**							
Age (per 10 years of increase)		1.29	0.75–2.23	0.36			
Sex							
Male	10/244	1		0.017	1		0.023
Female	12/108	2.93	1.22–7.00		2.85	1.15–7.05	
Year of visit							
1985–1992	13/125	1		0.063			
1993–1998	4/114	0.31	0.10–1.00				
1999–2005	5/113	0.40	0.14–1.16				
Nationality							
Dutch	19/305	1		0.97			
Non-Dutch	3/47	0.97	0.28–3.43				
Years of education after primary school							
<3	3/31	1		0.36			
3	4/35	1.20	0.25–5.86				
4–5	3/78	0.37	0.071–1.96				
>5	3/69	0.42	0.081–2.23				
Alcohol use in the past 6 months							
No	12/139	1		0.38			
Yes	9/151	0.67	0.27–1.64				
**Drug use related risk factors**							
Main type of noninjecting drug used in past 6 months							
Heroin	6/143	1		0.32			
Cocaine	13/154	2.11	0.78–5.70				
Cocktail of heroin/cocaine (i.e. speedball)	1/15	1.52	0.17–13.5				
Frequency of noninjecting drug use (main drug used) in past 6 months							
Multiple times daily	11/137	1		0.70			
Once daily	1/20	0.60	0.07–4.94				
Several times weekly, but less than daily	5/113	0.53	0.18–1.57				
Several times monthly, but less than weekly	1/20	0.60	0.07–4.94				
Once monthly	1/4	3.81	0.37–39.9				
Less frequent	1/11	1.14	0.13–9.79				
Non-injecting drug use of steady partner							
Not applicable, no steady partner	13/169	1		0.35			
No, never	4/35	1.55	0.47–5.06				
Yes, now or ever	4/91	0.55	0.17–1.74				
Start of injecting drug use during follow up							
No	15/305	1		0.02	1		0.043
Yes	7/47	3.38	1.30–8.80		2.78	1.03–7.47	
Years of regular heroin use							
Less than 6 months (or never start)	1/49	1		0.10			
6 months–5 years	3/66	2.29	0.23–22.6				
≥5 years	16/170	4.99	0.64–38.6				
Years of regular amphetamines use							
Less than 6 months (or never start)	18/242	1		0.59			
6 months or more	2/43	0.67	0.15–3.02				
**Cocaine related risk factors**							
Years of regular cocaine use							
Less than 6 months (or never start)	3/45	1		0.61			
6 months–5 years	6/112	0.79	0.19–3.32				
≥5 years	11/128	1.31	0.35–4.95				
Frequency of cocaine use in 6 months before ACS entry							
No cocaine use	1/38	1		0.45			
Once or more times monthly	1/28	1.37	0.082–22.9				
Once or more times weekly	5/87	2.26	0.25–20.0				
Once or more times daily	6/61	4.04	0.47–34.9				
**Sexual risk behaviour**							
Sex with injecting DU since 1980							
No	8/149	1		0.59			
Yes	4/54	1.41	0.41–4.89				
Sex with commercial sex workers since 1980							
No	5/94	1		0.80			
Yes	7/114	1.16	0.36–3.80				
Sex with MSM since 1980							
No	8/172	1		0.16			
Yes	4/36	2.56	0.73–9.02				
Main sexual preference since 1980 (excluding contacts with commercial sex workers)							
Exclusively heterosexual	15/285	1		0.18			
Not exclusively heterosexual	5/47	2.14	0.74–6.20				
Number of prostitution contacts in the 6 months preceding ACS entry (males and females)							
No prostitution contacts	1/20	1		0.76			
1–9	10/159	1.27	0.15–10.5				
≥10	8/95	1.75	0.21–14.8				
Prostitution contacts in the 6 months preceding ACS entry (males and females)							
No	8/155	1		0.49			
Yes	4/51	1.56	0.45–5.43				
Steady partner in the 6 months preceding ACS entry							
No	13/202	1		0.96			
Yes	9/137	1.02	0.42–2.46				
Steady partner that injects/injected drugs in the 6 months preceding ACS entry							
Steady partner injects/injected drugs	2/35	1		0.98			
Steady partner does/did not inject drugs	7/105	1.18	0.23–5.96				
Not applicable, no steady partner	13/202	1.13	0.24–5.26				
Last HIV test result of steady partner							
Not applicable, no steady partner in the 6 months preceding ACS entry	20/283	1		0.88			
Positive	1/10	1.46	0.18–12.1				
Negative	0/37	–	–				
Unknown	1/20	0.69	0.088–5.44				
Always use of condoms with steady partner							
Not applicable, no steady partner in the 6 months preceding ACS entry	2/15	1		0.33			
No	17/254	0.47	0.097–2.24				
Yes	3/83	0.24	0.037–1.60				
Always use of condoms with casual partners							
Not applicable, no casual partners in the 6 months preceding ACS entry	1/51	1		0.07			
No	18/212	4.64	0.60–35.6				
Yes	3/89	1.74	0.18–17.2				
Use of condoms with prostitution partners							
Always use of condoms	3/36	1		0.47			
Not always use of condoms	3/26	1.43	0.27–7.75				
Not applicable, no prostitution partners	16/289	0.64	0.18–2.33				
**Other risk factors**							
HIV status							
Negative	19/338	1		0.053	1		0.026
Positive	3/14	4.58	1.18–17.8		5.07	1.21–21.3	
Tattoo (ever)							
No	6/97	1		0.77			
Yes	8/111	1.18	0.39–3.52				
Piercing (ever)							
No	12/186	1		0.65			
Yes	2/22	1.45	0.30–6.95				
Jaundice (ever)							
No	6/70	1		0.11			
Yes	2/6	5.33	0.80–35.4				
Blood transfusion (ever)							
No	6/68	1		0.16			
Yes	2/7	4.13	0.66–26.1				

OR = odds ratio, 95% CI = 95% confidence interval.

In multivariate logistic regression, HIV-positive status (OR 5.07, 95% CI 1.21–21.3), female sex (OR 2.85, 95% CI 1.15–7.05) and starting injection during follow-up in ACS (OR 2.78, 95% CI 1.03–7.47), were independently associated with the presence of HCV antibodies.

### HCV RNA and phylogenetic analysis

**Fig. 1:**
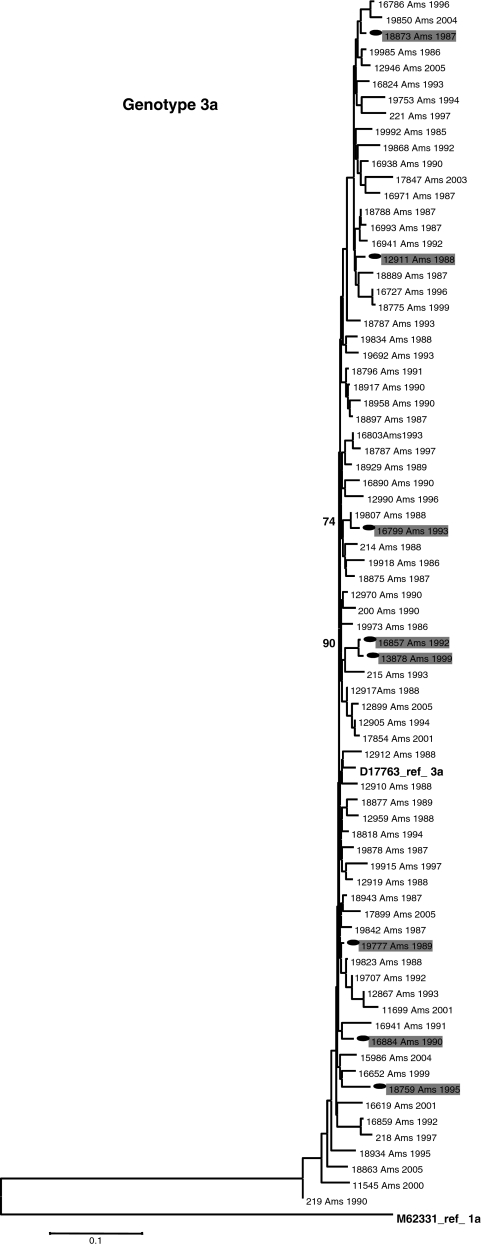
NS5B Phylogenetic tree of prevalent HCV genotype 3a infections among never-injecting drug users (DU) (shaded) and ever-injecting DU in Amsterdam, using the neighbour-joining method based on Tamura-Nei substitution with γ-distribution (α = 0.40). Each isolate code contains the year of sampling.

Of 22 HCV-antibody positive never-injecting DU at ACS entry, 15 (68.2%) had detectable HCV RNA. The most frequent HCV genotype found was 3a (53.3%), followed by genotype 1a (26.7%) (Table 1). HCV genotypes 1a and 3a are generally associated with injecting drug use, and in injecting DU in the ACS they account for 252/317 (79%) of HCV infections for which genotyping was performed. Hence, the proportion of injection-related HCV genotypes was comparable among injecting DU and never-injecting DU ([11,14], unpublished data). Figure 1 shows a phylogenetic tree of HCV genotype 3a, comprising the 8 NS5B sequences obtained from never-injecting DU together with all available genotype 3a NS5B sequences (*n*=65) from injecting DU ([11,14], unpublished data). Comparable to a pedigree, a phylogenetic tree illustrates the evolutionary relationships between genes or organisms or, in our case, the relationship among aligned NS5B sequences of several HCV genotype 3a viral variants. The more related two sequences are, the smaller the horizontal distance between those sequences in the tree. Based on phylogenetic analysis, sequences from never-injecting DU could not be distinguished from those of injecting DU. Sequences derived from never-injecting DU were interspersed with those of injecting DU, and they were not distinct phylogenetic isolates, nor did they form separate never-injecting DU clusters. This was observed also in HCV genotype 1a sequences (data not shown). The 3 never-injecting DU not infected with HCV genotype 1a or 3a harboured distinct strains of genotype 1b and 2a, which in the Netherlands and Belgium are linked to blood transfusion and nosocomial transmission rather than injecting drug use [15,16]. The proportion of never-injecting DU infected with these types (20%) was somewhat larger than the proportion observed among injecting DU (9%) in the ACS, but the difference was not statistically significant (*P*=0.26, Pearson chi-square).

Interestingly, only one never-injecting (male) DU seroconverted during follow-up despite denying injecting drug use. He has regularly reported a steady sexual relationship with an injecting (female) DU who also participates in the ACS. She is a known injecting DU and became chronically infected with HCV genotype 2b at least 2.7 years before her male never-injecting DU sexual partner seroconverted for HCV. When comparing their two HCV sequences, the sequences were 100% identical (data not shown), making accidental exposure during household contacts or sexual transmission the likely route of transmission in this couple.

## Discussion

In this cohort of never-injecting DU, the HCV prevalence was 6.3% (95% CI 3.7–8.8%). Although much lower than the prevalence in injecting DU in the same cohort (83.5%, [10]), this is substantially higher than in the general population in The Netherlands (estimated to be 0.1–0.4%, [17]). In literature, the HCV prevalence in never-injecting DU ranges from 2.3 to 35.3% [8]. However many studies were not specifically designed to measure HCV prevalence in never-injecting DU and often did not include questions on noninjection drug use risk factors for HCV.

The observed HCV incidence was very low at 0.049/100 PY, sixteen-fold lower than the HCV incidence estimated from the prevalent cases at study entry (0.79/100 PY). This suggests underreporting of past injecting drug use, which may have led to misclassification of injecting DU as never-injecting DU. However, this estimated HCV incidence has limitations: it does not take into account losses to follow-up in the unknown cohort that the prevalence sample is supposed to represent. Nor does it take differential recruitment of rates of healthy and infected subjects into account. However, when interpreted with caution, it could support our hypothesis of underreporting of injection drug use. Especially when injecting was incidental or stopped before entry in the ACS, participants may deny past risk behaviour, as has been described for HCV-positive blood donors in the Netherlands [15].

Starting injection later during follow-up was independently associated with a higher prevalence of HCV antibodies at entry. Of 352 never-injecting DU, 47 switched to injecting drug use after a median of 56 months (IQR 20–58 months). Of the 47, seven were among the 22 found to be HCV seroprevalent at entry. Again, this finding could suggest that some injecting DU were misclassified as never-injecting DU. They might have given socially desirable answers and denied injecting, since it is perceived among DU as damaging to their appearance and as overstepping a limit in the drug-using scene in Amsterdam [18]. Alternatively, the DU who started injecting during follow-up were already actively participating in the scene of injecting DU and were therefore more likely to become exposed to HCV through routes other than injecting drug use, such as needle stick accidents. Since HIV and HCV share transmission routes, the finding that HIV-positive never-injecting DU had a higher HCV prevalence at entry compared to HIV-negative participants, could imply that HIV-positive status is an indicator of unreported injecting drug use. On the other hand, HIV is transmitted sexually much more efficiently than HCV, and HCV might be transmitted more easily to and/or from HIV-positive individuals, compared to HIV-negative individuals, since HIV co-infection is associated with higher HCV RNA viral load [19].

Phylogenetic analysis revealed that the HCV sequences of never-injecting DU did not cluster together, suggesting that they were not a uniform group that became infected through sharing of noninjection drug use paraphernalia. In contrast, the noninjecting DU clustered together with the sequences found in injecting DU in the ACS (Fig. 1), indicating that they have close links with injecting DU and possibly underreport injection drug use. So although these DU did not report injecting drug use, they were infected from the pool of injecting DU (Fig. 1). Although self-reported data on methadone prescription in this cohort have been investigated and shown to be consistent with data from the Dutch Central Methadone Registration, self-reported data on sexually transmitted diseases (STD) were shown to be less consistent with diagnosis of such diseases [20,21]. In this study, based on the findings from logistic regression and phylogenetic analysis, some misclassification of ever-injecting DU seems likely in this never-injecting DU population.

Female sex was also associated with a higher HCV prevalence at entry, possibly indicating that women having sex with an HCV-positive partner are at higher risk for sexual transmission than men, as has been shown for HIV [22,23]. However, this gender difference has not yet been described for HCV [24]. We did not find an association between the presence of HCV antibodies and sexual behaviour. Furthermore, we observed only one HCV seroconversion during >2000 PY of follow-up, indicating that the risk of sexual transmission – and also household transmission-- is very small as has been demonstrated in partner studies among discordant heterosexual couples [25,26]. Unfortunately we were not able to perform risk factor analysis based on just one HCV seroconversion, but such analysis of incident cases in a longitudinal study would be more robust than a cross-sectional analysis of prevalent cases.

HCV has been detected on drug-use paraphernalia, and it has been hypothesized that HCV can be transmitted via these utilities (e.g. straws used for cocaine snorting) [27]. In line with our phylogenetic finding of nonclustering of never-injecting DU, we did not find statistically significant associations between cocaine use and the presence of HCV antibodies. However, questions on snorting paraphernalia were not included in the ACS questionnaires used in our study period. Some questions (e.g. having a tattoo, having a piercing) were added to the questionnaires in 2001 and thus yield data for only a portion of participants included in this study. A similar limitation holds true for the data on having received a blood transfusion, a question not asked after 1989, shortly before HCV screening of donor blood was introduced in developed countries. Moreover, never-injecting DU might potentially have received a blood transfusion when travelling to countries where transfusion is not yet safe. Although the direction of the effect of having received a blood transfusion was as expected (i.e. higher risk for those who have received a blood transfusion compared to those who did not), the main HCV genotype related to transmission by blood transfusion is genotype 1b, whereas the main genotypes circulating among never-injecting and injecting DU are 1a and 3a. Remarkably, in The Netherlands between 1997 and 2002, genotypes 1a and 3a, were found in 9/18 (50%) of HCV RNA-positive new donor candidates who most likely acquired HCV through a contaminated blood transfusion in the past [15].

In conclusion, although the incidence of HCV was very low in this study among never-injecting DU, the prevalence was much higher than in the general population. In the methadone outposts of the Amsterdam Health Service, HCV screening is offered every year irrespective of recent injecting drug use. Although, we could not distinguish whether the increased risk of HCV infection in never-injecting DU was related to underreporting of injection or to household or sexual transmission, HCV strains of never-injecting DU cluster with those found among injecting DU. HCV treatment has improved substantially since 2000 and is effective in up to 80–90% of patients [3]. Therefore, whatever the route of transmission, it is clear that routine HCV testing and treatment should be extended to both never-injecting and injecting DU.

## Abbreviations:

ACS: Amsterdam Cohort Study
DU: drug users
ELISA: enzyme linked immunosorbent assays
HCV: hepatitis C virus
IQR: interquartile range
PY: person years
RT-PCR: reverse-transcription polymerase chain reaction
